# Neuronal nitric oxide synthase in hypertension – an update

**DOI:** 10.1186/s40885-016-0055-8

**Published:** 2016-11-03

**Authors:** Yin Hua Zhang

**Affiliations:** 1 Department of Physiology & Biomedical Sciences, Ischemic/Hypoxic Disease Institute, Seoul National University, College of Medicine, 103 Dae Hak Ro, Chong No Gu, 110-799 Seoul Korea; 2 Yanbian University Hospital, Yanji, Jilin Province 133000 China; 3 Division of Cardiovascular Sciences, School of Medical Sciences, Faculty of Biology, Medicine and Health, University of Manchester, Manchester, UK

**Keywords:** Hypertension, Nitric oxide, Neuronal nitric oxide synthase (nNOS), Cardiomyocyte, Hypertrophy

## Abstract

Hypertension is a prevalent condition worldwide and is the key risk factor for fatal cardiovascular complications, such as stroke, sudden cardiac death and heart failure. Reduced bioavailability of nitric oxide (NO) in the endothelium is an important precursor for impaired vasodilation and hypertension. In the heart, NO deficiency deteriorates the adverse consequences of pressure-overload and causes cardiac hypertrophy, fibrosis and myocardial infarction which lead to fatal heart failure and sudden cardiac death. Recent consensus is that both endothelial and neuronal nitric oxide synthases (eNOS or NOS3 and nNOS or NOS1) are the constitutive sources of NO in the myocardium. Between the two, nNOS is the predominant isoform of NOS that controls intracellular Ca^2+^ homeostasis, myocyte contraction, relaxation and signaling pathways including nitroso-redox balance. Notably, our recent research indicates that cardiac eNOS protein is reduced but nNOS protein expression and activity are increased in hypertension. Furthermore, nNOS is induced by the interplay between angiotensin II (Ang II) type 1 receptor (AT1R) and Ang II type 2 receptor (AT2R), mediated by NADPH oxidase and reactive oxygen species (ROS)-dependent eNOS activity in cardiac myocytes. nNOS, *in turn*, protects the heart from pathogenesis *via* positive lusitropy in hypertension. Soluble guanylate cyclase (sGC)-cGMP/PKG-dependent phosphorylation of myofilament proteins are novel targets of nNOS in hypertensive myocardium. In this short review, we will endeavor to overview new findings of the up-stream and downstream regulation of cardiac nNOS in hypertension, shed light on the underlying mechanisms which may be of therapeutic value in hypertensive cardiomyopathy.

## Background

According to World Health Organization, over 1 billion people are associated with hypertension worldwide and the number of subjects with such disorder rises significantly in aged group [1–4]. Importantly, population with hypertension is in an upsurge in young generation. Attention should be drawn to this trend because the prevalent exposure to the risk factors for hypertension during early stage of the lifespan is likely to expend to the trajectory of later life of their adulthood [5] (“programming” for hypertension). Furthermore, hypertension-associated complication in the cardiovascular system is one of the most important causes of premature death worldwide (up to 45 % of death due to heart diseases). As such, the global economic burden of healthcare expenditure due to hypertension maintains a steady increase. So far, effective regime for hypertension or hypertension-related therapy for heart diseases is lacking.

Sustained pressure-overload in conjunction with systematic inflammation, oxidative stress and endothelial dysfunction in hypertension stimulates a series of adverse outcomes of the heart, including ventricular hypertrophy [6], collagen deposition or fibrosis [7], dilatation [8] and coronary artery stiffening or rarefaction [9, 10]. The fundamental mechanisms those cause these changes involve altered ion channels and transporters in the plasmalemmal membrane of cardiac myocytes; abnormal calcium handling in cardiac myocytes; metabolic derangement; sarcomere disorganization and changes in the wide spectrum of intracellular signaling pathways [6, 11–17]*.* Concomitant to the progression of hypertension-induced adverse remodeling of myocardium, a number of endogenous *defense* mechanisms can be triggered, which are involved in delaying and preventing the pathological processes. These defense mechanisms include: maintaining or stimulating cGMP pathway, e.g. nitric oxide [18], atrial natriuretic peptide [19, 20] and phosphodiesterase 5 inhibition [21]; antagonizing beta1-adrenergic pathway [22], e.g. beta3-adrenoreceptor- or muscarinic receptor dependent signaling pathways; AT2R, Ang 1–7 or 1–9 receptors and Mas receptor that antagonizes AT1R signaling [23–25]; metabolisms and AMPK signaling [26]. General consensus is that nNOS is an important cardiac protector in healthy and diseased hearts. In this review, we will highlight our novel findings in nNOS and its protective mechanisms of the heart in hypertension.

### NO, eNOS and nNOS in the hypertensive myocardium

NO is an important autocrine and paracrine signaling molecule that plays crucial roles in regulating cardiovascular physiology and pathology. Deficiency in NO are associated with oxidative stress, diastolic dysfunction and most of adverse cardiovascular disorders including heart failure and cardiac arrhythmias [18]. Two constitutive NO synthases (cNOS) are responsible for the production of NO in healthy myocardium: eNOS and nNOS, which share similar structure and mechanism of activation.

**Fig. 1:**
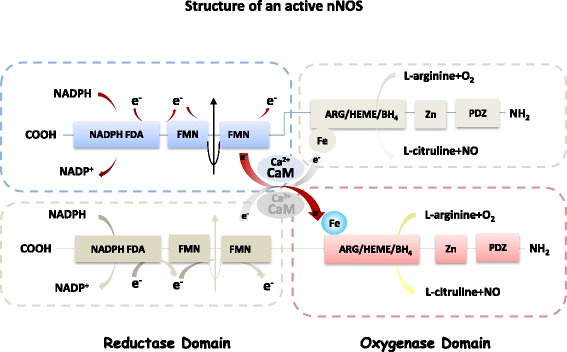
Structure of an active nNOS protein. Schematic diagram shows the two monomers of nNOS (each contains an oxygenase domain (−COOH terminal) and a reductase domain (−NH2 terminal). Electrons transfer from NADPH in the reductase domain of one monomar (*via* FDA and FMN) to the heme iron in the oxygenase domain of the other monomer, facilitated by calmodulin (CaM), enable nNOS to catalyze the oxidation of L-arginine to L-citrulline and releases NO. Ca^2+^ activates CaM and nNOS activity

In general, the active NOSs form a homodimer and convert the amino acid L-arginine to L-citrulline and NO (Fig. 1). NOS monomer contains a C-terminal reductase domain and a N-terminal oxygenase domain which are linked by calmodulin (CaM) binding region. The N-terminal oxidase domain contains the heme, tetrahydrobiopterin cofactors (BH4) and the binding site for the substrate arginine. Oxidase domain is the active site of NO synthesis. The reductase domain is composed of the flavin mononucleotide (FMN)-binding subdomain, the flavin adenine dinucleotide (FAD) and reduced nicotinamide-adenine dinucleotide phosphate (NADPH)-binding subdomains. During catalysis, the electrons are transferred from NADPH to FAD and FMN of one monomer to the heme domain of the opposite monomer [27]. Production of NO requires oxygen as the electron acceptor. NO diffuses freely across the plasma membrane, therefore, NO is generally known to be able to transport to the effector proteins in the cells or in the adjacent cells and exerts its effects (e.g. endothelial NO targets soluble guanylate cyclase, sGC, in smooth muscle to accomplish vasodilation).

In fact, NO is highly reactive with short life-time (a few seconds). Therefore, the likelihood of NO to travel a distance to reach the target proteins in myocardium is low (especially given the *oxidative* environment of myocardium); instead, NO is produced from eNOS and nNOS in the designated compartments and affect downstream targets in confined location where the enzymes reside [27, 28]. The spatial regulation enhances the effectiveness of NO and the producing enzymes to fulfill specific tasks.

It should be noted that eNOS and nNOS are localized at discrete compartments and exert diverse functions in cardiomyocytes. E.g. eNOS is localized in the caveolae of cardiomyocytes and nNOS is in the cytosol with ryanodine receptors (RyR) in the sarcoplasmic reticulum (SR) in healthy heart. The clear distinction in compartmentalization between eNOS and nNOS are disarranged in diseased hearts. E.g. eNOS expression is often found to be diminished and nNOS translocates from SR to plasmalemmal membrane [29–31]. The changes in the location of nNOS enable it to affect different downstream targets for different functions.

The pathological stimuli may also change the property of eNOS or nNOS in the hypertension. eNOS has been found to be uncoupled in the myocardium of a mice model of transverse aortic constriction (TAC)-induced pressure overload and eNOS produces superoxide rather than NO [32–34]. Increased oxidative stress and depletion of BH4 due to the oxidation of BH_4_ to BH_2_ is one of the dominant mechanisms for eNOS uncoupling, consequently, eNOS becomes responsible for cardiac oxidative stress, left ventricular (LV) hypertrophy and dysfunction [32–34]. In its “coupled” state, eNOS-derived NO can be an important protective mechanism for TAC-induced LV hypertrophy [35–38]. In addition, eNOS is known to be an “endogenous beta-blocker” by restoring the sympatho-vagal balance, promoting vasodilatation and neoangiogenesis [39] and protects the heart under pathological stress.

In contrast, nNOS has consistently been shown to be upregulated in LV myocardium from the hearts of pressure-overload [40, 41]. Similarly, the activity of nNOS (phosphorylated:total nNOS ratio) is significantly increased in right ventricular (RV) myocardium of pulmonary hypertensive rats [42], confirming the upregulation of myocardial nNOS with increased pressure-overload. Accumulating evidence shows that nNOS up-regulation in diseased heart is cardiac protective: nNOS has been shown to inhibit xanthine oxidoreductase [43, 44] or NADPH oxidases [45, 46] and mitochondria production of ROS [47], as a result, reduces oxidative stress and suppresses adverse myocardial remodeling. In addition, nNOS facilitates the relaxation of LV myocytes and maintains lusitropy of the heart under pressure-overload. Since increased oxidative stress and diastolic dysfunction are often associated with structural and functional pathology of the myocardium, such as hypertrophy, arrhythmias and heart failure, nNOS modulation of various cardiac oxidases or improving diastolic function of the myocardium under pressure-overload enable nNOS to protect the heart from structural and functional remodeling those underlie fatal heart failure.

### nNOS protein expression and activity in hypertensive myocardium

The mechanisms leading to nNOS up-regulation in diseased heart remain unclear. One of the suggestions is that the transcription and the translation of nNOS are oxygen-dependent. Hypoxia (8 %, 48 h) has convincingly show to increase the mRNA and protein expressions as well as the activity of nNOS in the systematic and local arteries (aorta, mesenteric arterioles and renal papilla) and in the brain by revealing a novel promotor region in exon 2 with increased translational efficiency [48]. Whether limited availability of O_2_ in hypertensive myocardium is essential in triggering nNOS up-regulation has not been elucidated yet.

**Fig. 2:**
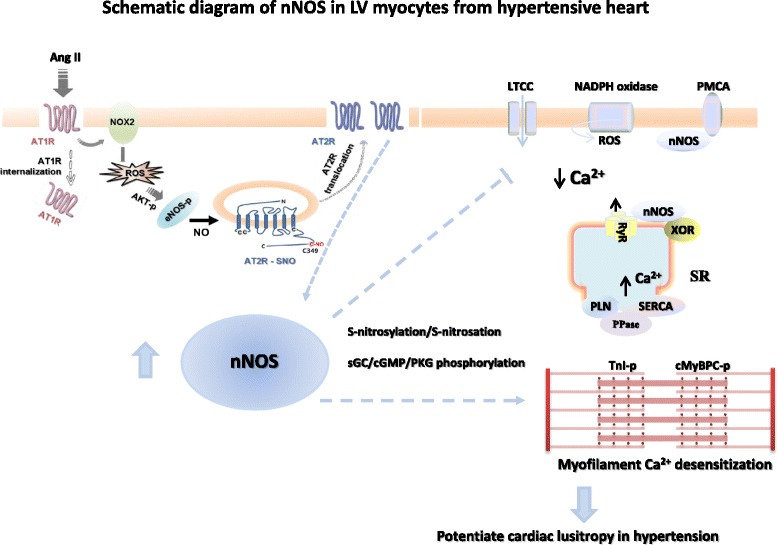
Schematic diagram of nNOS up-regulation by Ang II in cardiac myocytes and the potential target proteins, mechanism of regulation in cardiac myocytes from hypertension. *Left*. Ang II stimulates AT1R and NADPH oxidase in cardiac myocytes. AT1R and intracellular ROS activates Akt and phosphorylates endogeneous eNOS to produce NO. eNOS and NO-dependent S-nitrosation of AT2R lead to the translocation of AT2R to the plasma membrane and induces nNOS protein and increases NO production (data modified from Figure 8 of Basic Research in Cardiology, 2015, 110 (3):21). In hypertension, cardiac nNOS is up-regulated and facilitates myocyte relaxation. nNOS reduces Ca^2+^ influx *via* LTCC and promotes Ca^2+^ re-uptake *via* SERCA through PLN phosphorylation (secondary to PPase-dependent PKA phosphorylation) in healthy heart. In hypertension, nNOS inhibits LTCC and phosphorylates cMBP-C Ser^273^ and cTnI Ser^23/24^ *via* cGMP/PKG-dependent mechanism. As a result, myofilament Ca^2+^ sensitivity is reduced which accounts for nNOS-dependent positive lusitropy in hypertension

Intriguingly, our recent results demonstrated that Ang II induces nNOS under the conditions where ROS are increased (Fig. 2). In isolated LV myocytes, Ang II treatment (3 h) significantly increased nNOS protein expression and activity *via* AT1R stimulation of NADPH oxidase activity and ROS production [45, 49]. In addition, nNOS in LV myocytes is increased in Ang II-induced hypertensive rats [50] (Fig. 2). In both cases, Ang II only stimulated nNOS but not eNOS because eNOS protein expression is either not changed (with acute Ang II treatment) or significantly reduced (in hypertension) [45, 50]. This is in contrast to the findings shown previously where Ang II treatment in vivo increased eNOS protein expression in the myocardium [51, 52]. Therefore, Ang II or AT1R has been implicated in the activation of constitutive NOS in cardiovascular system.

Mechanistic studies showed that NADPH oxidase is essential in mediating AT1R-dependent and ROS-stimulated signaling (Fig. 2). NADPH oxidase/ROS activates eNOS-phosphorylation and mediates eNOS-derived NO-dependent S-nitrosation of AT2R. Site specific mutagenesis analysis indicated that among 4 intracellular cysteine residues in AT2R, cysteine349 of AT2R is the key regulatory site for AT2R translocation to the plasma membrane [49]. Our results are in line with other reports showing the relationship between AT2R and nNOS. E.g. C21, an AT2R agonist, significantly increased nNOS expression in the paraventricular hypothalamic nucleus (PVN) and rostral ventrolateral medulla (RVLM) [53, 54] or inhibition of AT2R abolishes relaxin-induced nNOS protein expression and phosphorylation [55]. Furthermore, nNOS is unaffected in the infarcted myocardium from AT2R gene deficient mice [56], confirming the role of AT2R in nNOS up-regulation. In hypertensive myocardium, protein expressions of AT1R and AT2R are not changed comparing to those in sham rats (unpublished data). It is possible to extrapolate that ROS may function as an important trigger signal to nNOS up-regulation at least at early stage of myocardial stress. Subsequent eNOS/NO-dependent S-nitrosation of AT2R and its downstream signaling cascades are pivotal in the transcription and translation of nNOS proteins. Our results are the first to reveal the interplay between AT1R and AT2R or eNOS and AT2R in regulating nNOS protein expression in cardiac myocytes. Application of the knowledge to the therapeutic strategy towards hypertension and pressure-overload-induced adverse remodeling will be beneficial.

### *S*-nitrosylation *vs.* soluble guanylate cyclase – cGMP pathway of nNOS in hypertensive myocardium

It is generally considered that the primary signaling pathway of NO is through the activation of soluble guanylyl cyclase (sGC) leading to the production of cyclic guanosine monophosphate (cGMP) and protein kinase G (PKG)-dependent phosphorylation of downstream proteins. However, it should be noted that the prototypic mechanism of NO involves direct modifications of a diverse range of proteins by *S*-nitrosylation (or *S*-nitrosation) of cysteine residues [57]. It turns out that the reaction between NO and target proteins are highly specific, requires substantially low concentration of NO and the process is actively regulated. The high specificity of NO-protein modification is achieved by NOSs being part of the signaling mechanism in protein complexes, in which *S*-nitrosylation or *S*-nitrosothiol (SNO) interaction occurs at the sites within the vicinity of NOSs.

NO activation of sGC/cGMP for PKG-dependent signaling requires high concentrations of NO [58]. I.e. NO activates sGC moderately *via* a classical heme-dependent mechanism at low NO concentration. However, at high concentration of NO, the activity of sGC can be increased by the order of 2 *via* heme-independent but *via* direct NO-cysteine interaction of sGC. Therefore, the concentration of NO near the vicinity of target proteins determine whether sGC is activated and cGMP/PKG-dependent pathway mediates the responses of NOSs.

It is well established that under normal conditions and in healthy heart, endothelial NO induces the relaxation *via* cGMP/PKG-dependent phosphorylation of myosin phosphatases in smooth muscle. In cardiac myocytes, however, the effect of nNOS on myocyte relaxation is independent of cGMP/PKG-dependent signaling, rather it is mediated by stimulating Ca^2+^ reuptake through Ca^2+^ ATPase in the SR (SERCA) *via* PKA-dependent phosphorylation of phospholamban at Ser [16] (PLN-p) [59]. The increased PKA-dependent phosphorylation is through nNOS-dependent inhibition of protein phosphatase 2A (PP2A) activity [59].

Interestingly, in LV myocytes treated with Ang II or from hypertensive hearts, increased nNOS activity facilitated myocyte relaxation *via* cGMP/PKG-dependent phosphorylations of PLN or cGMP/PKG-dependent phosphorylations of myosin binding protein C (cMyBPC-Ser^273^) and troponin I (cTnI-Ser^23/24^) (Fig. 2). As a result, nNOS reduces myofilament Ca^2+^ sensitivity and promotes myocyte relaxation in hypertension [50]. Inhibition of PKA-dependent signaling failed to prevent the effect of nNOS under these conditions. These results suggest that the downstream mediating mechanisms of nNOS-derived NO are shifted from sGC/cGMP-independent and PKA-dependent signaling pathway in healthy heart to cGMP/PKG-dependent mechanism in hypertensive hearts, presumably due to the greater bioavailability of NO from nNOS (Fig. 2). Similarly, cGMP/PKG-dependent signaling has been suggested to mediate the functions of nNOS following conditional nNOS over-expression in the myocardium in mice or lentiviral over-expression of nNOS in rats [60, 61]. Conversely, reducing myocardial nNOS and its activity in PMCA4b transgenic mice [62] or in the dystrophin deficiency model [63] decreased myocyte cGMP levels.

Recently, Kass’s group have shown that there are two pools of sGC in cardiac myocytes (caveolin-enriched membrane and nonlipid raft) with the former pool producing 2–3 fold more cGMP following NO stimulation [64]. Since nNOS up-regulation is known to be associated with translocation from SR to caveolae in the heart under stress [29–31] it is possible to postulate that nNOS up-regulation in diseased hearts or in response to pathogenic stimuli, e.g. Ang II, is associated with its translocation to the location where sGC is reachable with enhanced efficiency to produce greater amount of cGMP.

Therefore, the primary mechanism that mediates the effect of nNOS is *S*-nitrosylation or *S*-nitrosation of the cysteine residues of target proteins. Subsequently, the genre of downstream target proteins of NO determines the form of regulation in the signaling cascade. E.g. under the conditions of increased nNOS protein expression and activity, NO stimulates sGC (through S-nitrosation) and initiates cGMP/PKG-dependent signaling pathway. Alternatively, regulation of cardiac oxidases is able to affect downstream target proteins (e.g. Ca^2+^ handling proteins) to propagate the redox and Ca^2+^ signals, respectively.

### Proteins targeted by nNOS in healthy and hypertensive heart

So far, the downstream target proteins of nNOS and its mode of regulation in healthy and diseased myocardium are not fully understood. Key excitation-contraction coupling proteins are known to be targeted by nNOS: L-type Ca^2+^ channels [65, 66], RyR [31], PLN [59], voltage-gated sodium channels (Nav1.5) [67] as well as structure proteins, such as syntrophin [67]. Recently, we have reported that myofilament proteins (cMyBPC and cTnI) are targeted by nNOS and nNOS-dependent alterations in the phosphorylation and change the myofilament Ca^2+^ sensitivity [50]. Although nNOS-dependent regulation of myofilament proteins is not clear at present, our preliminary results suggest that the myofilament proteins those are regulated by nNOS are different between sham and hypertension, implicating the significance of nNOS in regulating cardiac myofilament (Fig. 2).

The detailed signaling molecules those are targeted by nNOS and the differences in healthy and diseased hearts remain undetermined. Cardiac oxidases (including xanthine oxidoreductase, NADPH oxidase or mitochondrial cytochrome C oxidases or eNOS) are probably the key targets of nNOS in the heart under pressure-overload and regulation of which induces a plethora of responses involving oxidation, reduction, phosphorylation and various types of post-transcriptional regulations [68]. Other proteins such as sGC or PPs, whose activity are important in the hearts, are likely to play important roles mediating the effects of nNOS in hypertensive heart.

## Conclusion

Hypertension is a common condition that leads to fatal cardiovascular complications such as myocardial infarction, stroke, sudden cardiac death and heart failure. Myocardial hypertrophy by sustained pressure-overload is the adverse remodeling in hypertension that predisposes to the apoptosis and fibrosis, malignant consequences upon pathogenic stimuli. During the pathological progression in the myocardium, nNOS is up-regulated and NO-derived pathway is associated with the prevention of cardiac hypertrophy by targeting oxidative stress, hypertrophic pathways and abnormal Ca^2+^ handlings. Our recent research reveals some of the important mechanisms of the upstream and downstream regulation of nNOS in hypertensive myocardium. Our results highlight that AT1R, NADPH oxidase/ROS and AT2R are important up-stream regulators of the transcription of nNOS in the myocardium under pressure-overload. In addition, nNOS-derived NO leads to cGMP/PKG-dependent phosphorylation of downstream target proteins including those in the SR and in the myofilament. Results from our research suggest that nNOS regulation of myofilament proteins is important in facilitating relaxation of the heart in hypertension. Our results reveal novel targets those possesses important clinical value in preventing the adverse remodeling with pressure-overload in the heart.

## Abbreviations

Ang II: Angiotensin II
AT1R: Ang II type 1 receptor
BH4: Tetrahydrobiopterin
CaM: Calmodulin
cGMP: Cyclic guanosine monophosphate
cMyBPC: Myosin binding protein C
cNOS: Constitutive nitric oxide synthases
cTnI: Troponin I
eNOS: Endothelial nitric oxide synthases
FAD: Flavin adenine dinucleotide
FMN: Flavin mononucleotide
LV: Left ventricular
nNOS or NOS1: Neuronal nitric oxide synthases
NO: Nitric oxide
PKG: Protein kinase G
PP2A: Protein phosphatase 2A
RV: Right ventricular
RyR: Ryanodine receptors
SERCA: Ca^2+^ ATPase in the SR
sGC: Soluble guanylate cyclase
SNO: S-nitrosothiol
SR: Sarcoplasmic reticulum
TAC: Transverse aortic constriction
